# Impact of Parenting Style on Early Childhood Learning: Mediating Role of Parental Self-Efficacy

**DOI:** 10.3389/fpsyg.2022.928629

**Published:** 2022-06-30

**Authors:** Chuibin Kong, Fakhra Yasmin

**Affiliations:** ^1^Department of Education, Yunnan Normal University, Kunming, China; ^2^School of Education, South China Normal University, Guangzhou, China

**Keywords:** parenting style, learning outcome, parental self-efficacy, early childhood, China

## Abstract

The current study examined the impact of parental style on early childhood learning, as well as the role of parental self-efficacy (PSE) as a mediating factor. In the domains of education and psychology, it is increasingly recognized that parents have a considerable impact on their children’s learning and development. Purposive sampling was used and data was gathered over 3 months from school children’s parents. Hypotheses were tested using smart partial least squares-structural equation modeling (PLS-SEM v3.2.8) software. The findings of the present study reveal that an authoritative parenting style is positively associated with learning outcomes among Chinese students. Moreover, the mediating role of parental self-efficacy has been tested and proved to be a potential mediator between parental style and children’s learning outcomes. High PSE is linked to parents’ adoption of a variety of optimum parenting practices throughout childhood, including maternal sensitivity and responsiveness to children’s needs, warm and affectionate parental behavior, and monitoring. Hence, low PSE has been linked to coercive or harsh parenting as well as a proclivity to give up easily when faced with parental difficulties. In China, further study is needed on the relationship between parenting style, parental self-efficacy, and learning outcome. Future parenting programs could also focus on raising parents’ understanding of the need for both parents’ involvement in expressive activities and mentoring. This could help them boost their parenting self-efficacy even more. Lastly, the implications for parents, children, and teachers are discussed.

## Introduction

Recent studies on parental education reveal that researchers have mostly focused on mothers, and while many authors have proposed the systematic inclusion of fathers, few studies have done so (Giallo et al., 2013; Diniz et al., 2021). Despite increased acknowledgment of the critical role of fathers play in their children’s growth and learning, research on parental self-efficacy and parental involvement in children’s education has tended to neglect fathers (Tazouti and Jarlégan, 2019). In educational policy and research, the role of parental involvement in children’s education has become a central topic (Acar et al., 2021; Ribeiro et al., 2021). In this regard, for enhancing student achievement and eliminating educational disparities, many school reforms include initiatives to promote parental involvement (Lo et al., 2021). The role of family life and parenting styles have a significant impact on the development and maturation of early children. Therefore, parental self-efficacy is typically a goal of programs aimed at improving early life experiences in order to encourage healthy parenting practices. Besides that, parents’ self-efficacy views, according to research may be crucial to the parenting practices. There is an increased sensitivity to learned helplessness and, as a result, a lack of drive to address problems when parents have low self-efficacy (Qutaiba, 2011; Gindrich, 2021).

Family involvement refers to parents active participation in a variety of activities and behaviors that encourage their children’s early learning and development. A good example is Head Start school (Kook and Greenfield, 2021). This federal program teaches parents how to work with their children at home, involves parents in early intervention to improve learning outcomes for children (especially those who are poor and underachieving), and provides opportunities for parents to participate in school administration. Obviously, the parent aspect is important to Head Start (Ma et al., 2016). For children’s cognitive and language development, parent involvement in play, learning, and routine home activities is critical (Tan et al., 2022). In this regard, parental involvement in literacy activities such as reading and telling stories is well known to be beneficial to children’s linguistic and cognitive development in the preschool years, as well as long-term academic outcomes. Parent participation, on the other hand, involves a broader range of parent actions than simply reading to children and can include any activity that gives a learning or cognitive stimulation opportunity (Eijgermans et al., 2022).

The role parental involvement is also significant in children’s academic outcomes and have also been related to the provision of educational toys, answering inquiries, and engaging in dialog with them about their experiences. Furthermore, having access to a computer at home and living in a family with a medium to high level of engagement in out-of-home activities such as visiting libraries were linked to optimal developmental outcomes for children (Le et al., 2021). Given the importance of parental involvement in boosting children’s outcomes, it’s not unexpected that there’s been a lot of curiosity about what factors influence the kind and frequency of parents’ involvement in activities with their children. Reduced cognitive stimulation in the home has also been linked to single parenthood and insecure employment (Martin et al., 2022). Even though these results tell us a lot about how economic disadvantage affects parent involvement. Giallo et al. (2013) study reveal that less attention has been paid to the psychosocial characteristics of the parent, child, and family setting that may affect how much time parents spend with their children at home. Based on the above-mentioned literature and gaps there is a need to further explore the impact of various parenting styles on early childhood learning with parental self-efficacy as a mediator. Additionally existing studies have mostly focused on structural or socioeconomic variables, implying that parents with less education who are from a lower socioeconomic standing and are experiencing financial difficulties, are less responsive to their children and provide less learning stimulation. Therefore, the present study focus is to highlight the different parenting styles influence on early childhood learning outcome and how the role of parenting self-efficacy mediates between the two variables.

## Research Literature

### Supporting Theories

The present study is based on the following theories. According to Bandura’s self-efficacy theory (Weinberg et al., 1979), perceived self-efficacy is a major driver of activity choice, task effort expenditure, and task perseverance in the face of impediments. Self-efficacy, while not the primary predictor of behavior, plays a significant part in people’s decisions about how much effort to put in and how long to keep it up when confronted with stressful conditions (Clarke-Midura et al., 2019; Ran et al., 2022).

According to Vygotsky’s social interaction theory, social interaction between the child’s mind and caregiver is a vital key to the child’s cognitive development (Forman and Cazden, 1986). Every parent wishes best for their children, especially when it comes to their intellectual abilities, moral values, and character development. Many parents, on the other hand, are unaware that educating and caring for their children in an overly restricted or overly permissive manner might cause them to lose confidence and ambition to succeed. According to several researchers, the family environment, particularly parenting behavior, influences interpersonal competence and changes in development, including social academic achievement, in teenagers (Whittaker and Cowley, 2012; Shian et al., 2022). According to Grolnick and Ryan (1989), one factor that influences adolescent school competency is the familial environment. The family environment reveals several relationships between parents and children that have an impact on one another, particularly in terms of parental style. In this scenario, the family environment, in the form of parenting style, also provides a learning pattern and facility. Theresya et al. (2018) proposed that a good parenting style creates a positive emotional environment and boosts a child’s self-confidence while learning, which helps the child do better in school.

### Parenting Style and Learning Outcome

Early childhood and early school years have long been recognized as crucial to adult well-being and success (Diniz et al., 2021). Education that is developmentally appropriate from an early age leads to better educational outcomes later in life (Ma et al., 2016). Despite the large quantity of studies done in this area, there are major discrepancies in how parents are conceptualized and measured (Tazouti and Jarlégan, 2019). Some academics define parental involvement as involvement in school activities; others define it as parental ambitions for their children; and yet others define it as involvement in their children’s home learning activities. Researchers have recently acknowledged that the concept of parental participation is multidimensional (Dewi and Indrasa, 2017), encompassing a wide range of parental behaviors related to their children’s education. Epstein (1992) defined parental involvement as (1) parent practices that create a positive learning environment at home; (2) parent-school communications about school programs and student progress; (3) parent participation and volunteering at school; (4) parent and school communications about learning activities at home; (5) parent involvement in school decision making and governance; and (6) parent access to a school’s resources (Catsambis, 2001). Parents’ involvement in their children’s education, according to Epstein, is not static. Rather, differences in any of three overlapping domains of influence family, community, or school can alter the forms of parental participation (Epstein, 1992). Based on the previous literature, these variables were studied separately with either father or mother influence on children success. But the objective of present study was to explore the influence of both parents on children’s and how parental self-efficacy leads to better learning outcome (Zeb et al., 2021).

Children’s relationships with their families are critical to their growth (Popa, 2022). Parental child care attitudes are defined by the parents’ warm and caring approach to the child; expectations of the child; communication with the child; and disciplinary attitudes toward them. In family attitudes theory, Diana Baumrind identified three categories of parental attitudes: families that are permissive, authoritative, and authoritarian (Baumrind, 1968). Permissive parents take the strategy of tolerating and endorsing behavior based on the wishes of their children without looking into the causes or grounds of the behavior (Liu and Guo, 2010). Although the child’s behavior is harmful to the environment, it is tolerated, and the parents are powerless to encourage the child to follow the rules. While such parents have greater talents in terms of child care, they have less ability to control their children’s conduct. They give their children too much freedom, lack discipline, and have low expectations of their children (Verrocchio et al., 2015). Furthermore, authoritarian parents use strict rules and constraints formed by an excessive level of authority to control their children’s behavior. For those parents, what matters is that their children follow the rules without questioning them, and that their parents interfere and regulate their children’s behavior without hesitation for the sake of the child. Despite their failures in child care, these parents have the mindset of having the most parental control. They use both verbal and non-verbal (physical) sanctions to penalize the child’s unwanted behavior while failing to appreciate positive behavior (Song et al., 2022).

Moreover, in this parenting style parents place unrealistic expectations on their children (Ren and Zhu, 2022). These are the parents who are the most resistant to change and also make swift decisions. Lastly, with verbal and physical emotions authoritative parents assist their children. They have compassionated and close ties with their children. Those parents approach their children in a more cooperative manner. Their expectations are based on the abilities of their children. Those parents are attempting to mold their children’s cooperative and sensitive behavior. They are aware of their children’s thoughts, feelings, and attitudes, and they treat them with respect (Liu et al., 2022). The norms of authoritative parenting, which are widely regarded as the most ideal kind of parental care and attitudes, are open, obvious, and debatable. Because of its adaptable structure, it can be reconfigured.

The role of these parental attitudes and actions can have an impact on their children’s personality traits and adaption to their surroundings. Growing up in a family with permissive parents might make children selfish. These children are uninterested in other people’s feelings and thoughts. They may be lacking in self-control and have low self-esteem. They could be lacking in social skills. Anxiety, sadness, and uneasiness may be experienced by children of authoritarian parents. When they are furious, they may resort to more physical aggression. Furthermore, they are unable to communicate effectively. They may exhibit a lack of self-assurance. In social situations, they are introverted people who can be confrontational. Children raised by authoritative parents are more capable socially and accept responsibility; they are self-assured, cooperative, pleasant, cheerful, autonomous, socially skillful, and independent (Önder and Gülay, 2009).

With the growing emphasis on early childhood education and school success, it’s more important than ever to understand the development of skills, abilities, knowledge, and behaviors that are particularly important to children. When it comes to defining learning outcomes, there are two ways that are commonly used. One method is to identify and describe desirable learning outcomes for children at various developmental stages using crucial domains of child development. The five domains of learning and development for children in early childhood education and early primary education have been highlighted by the National Education Goals Panel as vital to enhancing human development. Physical well-being and motor development, social and emotional development, and learning approaches (learning styles) that include cultural components of learning, language development, and cognition and general knowledge are among these categories (Ma et al., 2016). The way parents raise their children has a big impact on their development and learning.

In western societies, research has consistently proven that parenting style has a direct relationship with children’s academic achievement (Luo et al., 2021). In general, research shows that children raised by authoritative parents have the best outcomes, whereas children raised by authoritarian or permissive parents have the worst outcomes. A study found that parenting style had a significant impact on children’s self-concept development. The reported level of warmth demonstrated by both their fathers and mothers had a direct relationship with the children’s self-concepts but not with parental permissiveness. Moreover, another previous study discovered that the family style affects the process of acquiring self-efficacy as outlined by Bandura (1986). According to previous studies focused on western cultures, authoritarian and permissive parenting styles have a negative impact on children’s academic achievement (Huang and Prochner, 2003). Therefore, Hypothesis 1 is constructed as shown below:

H1: Parenting style is positively associated with early childhood learning outcome.

### Parental Self-Efficacy and Learning Outcome

Parenthood, while frequently rewarding, is also fraught with stress-inducing obstacles. New parents must deal with the physical and financial demands of caring for a child, as well as the various lifestyle changes that might arise as a result of this additional duty and lead to negative consequences such as strained spousal relationships and social isolation (Song et al., 2022). The emotional cost of parents’ lack of confidence in their capacity to care for their children was noted as a concern for new parents as early as 1986. Once it was found, the idea of being confident in oneself and one’s skills as a parent was called parental self-efficacy, and it was immediately understood within a Bandura (1986) framework.

In the following decades, parental self-efficacy (PSE), which has lately been defined as “parents’ belief in their ability to influence their child in a health and success-promoting manner,” has emerged as a key treatment target for parent and child well-being. Parental self-efficacy has remained relevant in published literature since its inception as such an important clinical emphasis (Albanese et al., 2019). Parental self-efficacy research is based on Bandura’s (1986) self-efficacy theory, which states that one of the major processes influencing behavior is an individual’s conviction in their capacity to effectively complete a task or sequence of activities. As a result, PSE measures a parent’s ability to mobilize the cognitive resources and actions required to exert control over life events. While self-efficacy is defined as a dynamic dimension that varies depending on the task’s needs, external variables, and a person’s previous experiences (Tazouti and Jarlégan, 2019).

A parent’s job is a complex and hard opportunity to support and contribute to a child’s growth and development. In this regard, parental competence is made up of behavioral, affective, and cognitive elements, with parental self-efficacy being a key component. Coleman and Karraker (2000), for example, reported the following findings: parents with high self-efficacy believe they can effectively and positively influence their children’s development and behavior, and they engage in positive parenting behaviors, are more responsive to their children’s needs, engage in direct interactions with their children, use active coping strategies, and perceive their children to have fewer behavioral problems. On the other hand, for parents who have poor self-efficacy, the opposite is true. Previous literature supported that parent with low self-efficacy, for example, have higher rates of depression, exhibit more defensive and controlling behavior (Zeb et al., 2021), have higher perceptions of child difficulties, report higher stress levels, have a passive parental coping style, place a greater emphasis on relationship problems, show more negative affect, feel helpless in the role of parent, and use punitive disciplinary strategies (Pelletier and Brent, 2002).

Consequently, parents’ involvement in their children’s learning and academic progress is generally beneficial, according to researchers. Greenwood and Hickman (1991) identified a number of studies concentrating on primary school students that found links between parental participation and academic achievement, well-being, attendance, student attitude, homework readiness, grades, and educational goals. The findings revealed that academic achievement, time spent on homework, positive attitudes toward school, and lower rates of high school dropouts are all favorably associated with parental participation (Gonzalez-DeHass et al., 2005). Several studies have found that parents from higher socioeconomic backgrounds are more involved in their children’s education than parents from lower socioeconomic backgrounds, and that this involvement fosters more positive attitudes toward school, improves homework habits, reduces absenteeism and dropout, and improves academic achievement (Sui-Chu and Willms, 1996). Aside from the high emphasis put on education by Chinese parents, the fierce rivalry for a limited number of spots in higher education has an impact on parents’ parenting behavior. According to prior research performed in China, over 83 percent of parents said they helped their children study in various ways, such as hiring tutors or supervising their children’s homework (Zhang, 2021). In Hong Kong and Taiwan, studies on the relationship between Chinese parenting style and children’s results were also conducted (Luo et al., 2021). In another study, high self-esteem is linked to positive parent-child relationships among Chinese teenagers (Tan et al., 2022). Children who have a poor relationship with their parents, on the other hand, exhibit higher maladjustment and deviant behavior. According to a Taiwanese study (Huang and Prochner, 2003), low achievement motivation and bad learning attitudes were linked to rejecting and inconsistent parents. As a result, the following hypothesis has been developed:

H2: Parental self-efficacy is positively associated with early childhood learning outcome.

### Mediating Role of Parental Self-Efficacy Between Parenting Style and Learning Outcome

In the current study parental self- efficacy mediates between the relationship between parenting styles and learning outcome. Parental self-efficacy is another key theoretical construct for understanding influences on parental participation. It is a powerful predictor of parent behavior, with parents who feel more efficacious in their parenting position more likely to engage in parenting actions that are crucial in improving children’s social, emotional, and behavioral development (Zeb et al., 2021). High PSE is linked to parents’ use of a variety of optimal parenting strategies throughout childhood, including maternal sensitivity and responsiveness to children’s needs, warm and affectionate parenting behavior, and monitoring, according to comprehensive descriptive reviews. Low PSE, on the other hand, has been linked to coercive or harsh parenting as well as a proclivity to give up when faced with parental obstacles. While PSE has been associated to increased participation in home learning activities such as reading and helping with homework with older children. Previous research on the relationship between PSE and engagement in play, learning, and home activities with younger children is limited (Giallo et al., 2013).

Parental self-efficacy can have a direct impact on a child’s adaptive ability, but it can also have an indirect impact on a child’s adaptive capacity due to their parents’ engagement behavior. Parents with a high PSE score have fewer negative emotions and are more confident in dealing with challenging parenting situations, which benefits their children’s learning (Zeb et al., 2021). It is critical for children’s development that parents establish a cognitively stimulating home learning environment. According to previous research, PSE moderated the association between parents’ positive perceptions (e.g., individual teacher invitations and general school invitations) and children’s achievement. Parents’ self-efficacy also plays a mediating function in parents’ negative emotions (e.g., parental stress) and parenting practice behaviors, according to previous research, which can help to mitigate the detrimental impact of parents’ emotions on parenting practice behaviors (Liu et al., 2022).

**FIGURE 1:**
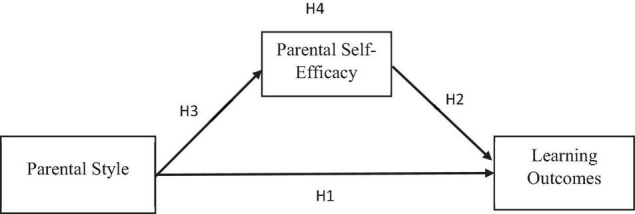
Conceptual model.

The purpose of this study was to determine the impact of parental participation on children’s learning outcomes among Chinese children, as well as the role of parental self-efficacy as a mediating factor. In the domains of education and psychology, it is increasingly recognized that parents have a considerable impact on their children’s learning and development. Academic achievement is very important to Chinese parents, and they expect their children to work hard in class. In this regard, parenting was defined as a series of actions and interactions between a parent and a child that had the potential to affect one another until the child reached adulthood. Parents were figures who played a vital role in the process of parenting, and they were obliged to continue to support and nurture their children’s growth, not just physically but also emotionally (Dewi and Indrasa, 2017). According to Martin and Colbert (1997), gender, childhood background, and parental beliefs are among the characteristics that can influence the parenting process. Gender influences the parenting process since it is assumed that moms and dads have a closer relationship. Another component determining parenting is childhood background, and the third factor is parental belief. According to Martin and Colbert (1997), beliefs are the most essential since they influence a parent’s values and behavior. Despite the fact that their confidence originates from nature, and their function as a parent has been influenced by their experiences since childhood, the shape and level of their confidence will change depending on how individuals perceive them (Martin and Colbert, 1997). As a result, the purpose of this study is to look into the impact of parenting style on learning outcomes among Chinese children, as well as the role of parenting self-efficacy as a mediator as presented in Figure 1. The above literature leads us to hypothesize that:

H3: Parenting style is positively associated with Parental self-efficacy.

H4: Parental self-efficacy mediates the relation between parental style and learning outcomes.

## Research Methodology

Self-administered questionnaires completed by presents of early child’s school students studied in different school located in China. The purposive sampling was used (Etikan et al., 2016), and data was gathered in 3 months, from January 2022 to March 2022. The data was gathered at a time, and therefore, the study is cross-sectional (Kesmodel, 2018). Author first gets the permission of their school boards and administrators, were used to collect data for this research. After class, students received a packet that included two surveys for their parents, as well as a cover letter. When the cover letter outlined the study’s purpose, to better understand the role of parents in children’s education it asked each parent whether they could complete their own questionnaire without consulting the other. Students returned the completed surveys for their parents to school.

According to G* power software, the minimum sample size necessary for this research is 119 respondents to achieve a power of 0.95 and a medium size effect of 0.15 (Faul et al., 2007). However, the researchers obtained data from 235 parents, exceeding the required sample size. A total of 290 surveys were distributed, and 250 parents returned the survey. After the deletion of incomplete responses, 235 surveys remained useable, representing an 81.03% response rate. The response rate was quite encouraging in the difficult Covid-19 pandemic situation. Out of the 235 parents, 150 mothers and 85 were fathers completed the surveys. Majority of parents age from 35- to 50 years, had a had a degree from a college or university, and household income more than $60,000 to $80,000 United States dollars.

The questionnaire consisted of a total of 58-items, including: a 30-items scale developed by Robinson et al. (1995) was used to measure parental style the independent variable, and a 20-items scale was used for dependent variable children’s learning outcomes adopted from Ajibade et al. (2020). For the mediating variable parental self-efficacy 8-items scale adopted from Liu and Leighton (2021). All scale evaluated based on five-point Likert scale, comprising 1 (strongly disagree), 2 (disagree), 3 (neutral), 4 (agree) and 5 (strongly agree).

**TABLE 1:** Measurement model.

Constructs/Items	Factor loadings	Cronbach’s Alpha	rho_A	CR	AVE	Source
Parental style		0.937	0.942	0.944	0.520	Robinson et al., 1995
Ps1	0.524					
Ps2	0.554					
Ps3	0.589					
Ps4	0.735					
Ps10	0.579					
Ps11	0.598					
Ps12	0.655					
Ps13	0.664					
Ps14	0.533					
Ps15	0.648					
Ps17	0.738					
Ps18	0.726					
Ps19	0.718					
Ps20	0.744					
Ps21	0.745					
Ps22	0.742					
Ps23	0.771					
Ps24	0.730					
Ps25	0.747					
Ps26	0.631					
Ps27	0.731					
Ps28	0.727					
Ps29	0.633					
Ps30	0.554					
Learning outcomes		0.936	0.945	0.944	0.564	Ajibade et al., 2020
Lo1	0.714					
Lo2	0.762					
Lo3	0.724					
Lo4	0.746					
Lo5	0.764					
Lo6	0.756					
Lo7	0.785					
Lo8	0.581					
Lo9	0.763					
Lo10	0.768					
Lo11	0.788					
Lo12	0.799					
Lo13	0.902					
Lo14	0.789					
Lo15	0.619					
Lo16	0.659					
Lo17	0.536					
Lo18	0.558					
Lo19	0.704					
Lo20	0.773					
Parental self-efficacy		0.812	0.863	0.865	0.570	Liu and Leighton, 2021
Pse1	0.821					
Pse2	0.811					
Pse3	0.816					
Pse4	0.794					
Pse5	0.832					
Pse6	0.707					
Pse7	0.695					
Pse8	0.729					

## Results

The analysis was conducted with the help of Smart PLS v.3.0 (Wong, 2013). The variables of the survey questionnaire are evaluated and the instrument is made accurate during the first phase of the measurement model. Based on the bootstrapping approach (*T*-tests for 5,000 sub- samples), Hair et al. (2017) determined whether or not factor loadings, weights, and path coefficients were statistically significant for each variable. Factor loadings assessments are carried out, as well as Cronbach’s Alpha, Composite Reliability (CR), and Average Variance Extracted (AVE) analyses. The validity of explicit indicator hypotheses may be determined by their factor loadings, which indicate that loadings greater than 0.50 on two or more variables are substantially reflected (Hair et al., 2011). As a result, the three variables and parental style, parental self-efficacy, and learning outcomes all provide valid measurements of their respective variables, as seen in Table 1. According to Hair et al. (2020), AVE value is must be greater than 0.5 and the CR and Cronbach’s Alpha are more than 0.6, then variable’s convergent validity is accepted. Hair et al. (2020) developed a strategy for excluding items with factor loadings between 0.40 and 0.70 from assessment if excluding observed variables increases AVE and composite reliability values in reflective scales. Thus, items PS 5, 6, 7, 8, 9 and 16 for parental style were deleted for the increased of AVE values. By deleting particular items, factor loadings, Cronbach’s Alpha, CR, and AVE calculations will exceed the recommended cut-off values. The Table 1 shows the measurement model that has a convergent validity.

Furthermore, the Henseler et al. (2015) proposed Heterotrait-Monotratit (HTMT) approach was applied. The discriminant validity of the HTMT approach was evaluated in two ways. To begin, the threshold value was determined using HTMT. A value greater than the HTMT threshold value demonstrates the absence of discriminatory validity. The precise HTMT cutoff value is controversial “when the correlation is near to one.” Some researchers have offered a threshold value of 0.85 (Ab Hamid et al., 2017), while others have suggested a value of 0.90 (Henseler, 2017). Second, discriminant validity was assessed and established by examining the confidence intervals around the HTMT values that were less than one. When the value 1 is removed from the interval range, it demonstrates that the variables are empirically clear. According to Table 2, the HTMT values for all constructs are less than 0.85. As a result, this research accepts discriminating validity.

**TABLE 2:** Discriminant validity through Heterotrait-Monotratit (HTMT).

Constructs	Learning outcomes	Parental style	Parental self-efficacy
Learning outcomes			
Parental style	0.780		
Parental self-efficacy	0.646	0.715	

After the measurement model has been calculated, the structural equation model of the observed data is constructed. With the use of bootstrapping technique, we were able to find significant correlations between the variables. We employed the method proposed by Henseler (2017), to investigate the relationships between the parental style and learning outcomes through mediating role of parental self-efficacy. Therefore, consequently, four particular criteria were utilized to examine the direct and indirect impacts of the structural equation model: To begin, we examine every construct. The degree of R^2^ for endogenous latent variables is used to estimate the variance for each construct (Hair et al., 2017). An adequate assessment of R^2^ may be conducted depending on the research arrangement (Cohen, 1988). High, medium, and low scores were calculated as follows: 0.26; 0.13; and 0.09. Despite this, the direct effect model in the current study has a 53.5% R^2^ value for the endogenous variables of the defined parental self-efficacy, which means that 53.5% of the change in parental self-efficacy is predicted by parental style. Moreover, R^2^ value for early childhood learning outcomes is 0.875, which suggests that 87.5% change of learning outcomes is predicted by parental style and parental self-efficacy. Therefore, Table 3 indicate, the model shows a reasonable predictive accuracy.

**TABLE 3:** Coefficient of determination in the PLS method.

Constructs	R Square	R Square Adjusted	Q^2^ (= 1-SSE/SSO)
Learning outcomes	0.875	0.874	0.398
Parental self-efficacy	0.535	0.533	0.243

Secondly, a cross-validation redundancy (Q^2^) was employed to evaluate the accuracy of the research model in identifying its significant aspects in order to establish predictive significance (Hair et al., 2017). As shown in Table 3, the direct impacts of each of the above-mentioned factors on early childhood learning outcomes are represented by Q^2^ = 0.398 and parental self-efficacy Q^2^ = 0.243, which indicates that the value of Q^2^ is greater than zero. Hence, the model’s appropriate predictive relevance can be considered (Henseler et al., 2015). The findings also support the direct hypotheses H1 H2 and to H3, the direct effect of parental style on early childhood learning outcomes is positive and significant (β = 0.129, *p* < 0.001), Furthermore, the direct effect of parental style on parental self-efficacy has positive and significant impact (β = 0.786, *p* < 0.000) and parental self-efficacy on learning outcomes (β = 0.824, *p* < 0.000), this suggests that the hypotheses H1, H2 and H3 have been accepted.

In addition, the effect size (f^2^) is the impact of an independent variable on the dependent variable to estimate the magnitude of an exogenous (independent variable) effect on the endogenous (dependent variable) (Hair et al., 2017). An effect size (f^2^) estimates between 0.02 and 0.15 or 0.35, according to Cohen (1988), reflects medium, small and large effects, respectively. Table 4 indicated the effect size as follows: 0.515 for PS to LO, 1.153 PS to PSE and 1.178 PSE to LO. The findings show that these exogenous factors have a medium and large impact on the endogenous variables, respectively. Finally, the Table 4 presented that the indirect mediating effect of parental self-efficacy on the relationship between parental style and learning outcomes is positive and significant (β = 0.648, *p* < 0.001). Therefore, hypothesis 4 is accepted.

**TABLE 4:** Results of the structural equations model.

Hypotheses	Relationship among constructs	β	M	S.D.	T Values	F Values	*P* values	Remarks
	**Direct effect**							
H1	PS - > LO	0.129	0.132	0.043	3.036	2.515	0.002**	Supported
H3	PS - > PSE	0.786	0.790	0.021	38.175	1.153	0.000***	Supported
H2	PSE - > LO	0.824	0.822	0.037	22.027	1.178	0.000***	Supported
	**Indirect effect**							
H4	PS - > PSE- > LO	0.786*0.824 = 0.648	0.650	0.036	18.177		0.000***	Supported

PS, parental style; LO, learning outcomes; PSE, parental self-efficacy; S.D., standard deviation. *p < 0.05, **p < 0.01, ***p < 0.001.

## Discussion

The current study looked into the effect of parental involvement on early childhood learning outcomes as well as the role of parental self-efficacy in mediating this effect. The academic engagement of students can be influenced by parental actions. Song et al. (2022) looked at how parent participation affected the social and academic functioning. Izzo et al. (1999) used a 3-year longitudinal design to examine several aspects of parent involvement, including the number of educator contacts with parents, the quality of those interactions, parents’ participation in school activities, and parents’ involvement in home activities to help their children develop socially and academically. Tan et al. (2022) studied how these parental involvement variables influenced students’ school engagement, which is particularly relevant to this topic. Students’ engagement was assessed by looking at their attention-getting activities, work habits, task orientation, operating in the face of distractions, frustration tolerance, and ability to cope with failure. Parents’ involvement in school activities was found to be a favorable predictor of student engagement. Surprisingly, higher levels of parent–teacher communication were linked to lower levels of school involvement (Gonzalez-DeHass et al., 2005). The parent involvement rating scale, parental sense of competence scale, and student assessment of learning gains scale were all employed in the study.

The following hypotheses had to be tested: First, there would be a significant link between parental involvement and learning outcomes. The findings demonstrated a strong, beneficial relationship between parental participation and learning outcomes. Parents who are more involved in their children’s activities at home and at school have higher results. According to research (Epstein, 1992), parental participation is positively associated with children’s achievement and motivation to learn. Previous research has demonstrated the significance of parents’ educational goals for their children. In both primary and secondary schools, high parental ambitions are closely linked to student accomplishment (Catsambis, 2001). Parental participation has been shown to improve students’ math proficiency and accomplishment, as well as advances in reading ability and performance on standardized examinations and academic evaluations. Furthermore, parental involvement has been linked to fewer behavioral issues at school, improved attendance and class preparation, course completion, and decreased dropout rates (Fan and Williams, 2010).

Second, there would be a significant relationship between parental self-efficacy and the learning outcome. The findings demonstrate a positive relationship between parental self-efficacy and learning outcomes. Parental self-efficacy is described as parents’ opinions of their abilities to positively impact their children’s behavior and development in the field of parenting. Parental self-efficacy can be characterized in terms of schooling as parents’ belief that they can have a positive impact on their children’s learning and academic accomplishment. PSE has been connected to parental educational methods, which have been extensively researched. According to previous research, mothers and fathers with strong parental self-efficacy are more involved in their children’s everyday learning and play activities. Several studies have found that when parents have high hopes and expectations for their children, they achieve higher academic achievements and stay in school longer than when their parents have low aspirations and expectations (Tazouti and Jarlégan, 2019).

Third, there would be a significant relationship between parental self-efficacy and parental involvement. According to research, parental self-efficacy is linked to a better knowledge of the role of parents and boosts parents’ monitoring of their children’s schooling. Parental self-efficacy, parental involvement in their children’s education, and academic accomplishment are also linked. PSE also predicts parental involvement and monitoring, which predicts adolescent academic adjustment. Previous research has demonstrated a strong relationship between PSE and parental involvement. Both parents had a deep connection, but the mother’s was stronger. There have been few previous studies that have looked for empirical correlations between these two ideas. According to a prior study, PSE is linked to parents’ knowledge of their role in their children’s education and leads to parents being actively involved in their children’s education (Tazouti and Jarlégan, 2019). Finally, there would be a considerable link between parental involvement and children’s learning outcomes, with parental self-efficacy serving as a mediating factor. The findings are consistent with earlier research. High PSE is linked to parents’ adoption of a variety of optimum parenting practices throughout childhood, including maternal sensitivity and responsiveness to children’s needs, warm and affectionate parental behavior, and monitoring, according to comprehensive descriptive studies. Low PSE, on the other side, has been linked to coercive or harsh parenting, as well as a proclivity to give up easily when faced with parental difficulties (Giallo et al., 2013). According to research, parents that have low parenting self-efficacy experience bad outcomes in their parenting. Low parenting self-efficacy was discovered to have a negative impact on parental behavior toward their children. According to the Indonesian Child Protection Commission (Setyawan, 2015), violence against children in Indonesia has increased over time, with the primary perpetrators being their own parents. The main reason for this was because parents felt they had failed and were no longer capable of caring for their children, so they vented their frustrations by using violence on their children when they made mistakes. In fact, research shows that parents with high parenting self-efficacy view parenting challenges as a challenge rather than a threat, which can lead to them harming their own children. Parental participation has a substantial association with parenting self-efficacy, according to previous study (Dewi and Indrasa, 2017).

## Conclusion

The purpose of the present study was to investigate the effect of parental involvement on children’s learning outcomes as well as the role of parental self-efficacy as a mediator. Findings of this study supported our hypothesis of parenting styles, learning outcomes and parental self-efficacy having a significant positive relation. The results revealed that parental participation had a considerable impact on learning outcomes in Chinese students. The study findings also affirm that there is a significant positive relation between parental self-efficacy and learning outcomes. From this study it can be inferred that those students who have parental involvement in their education are more likely to take personal responsibility for their education as compared to others. Besides that, students adopt a mastery goal orientation to learning when their parents show an interest in their child’s education by getting involved. Moreover, those parents who are not aware regarding the needs of students at educational level leads to negative impact on the children’s learning outcome. Therefore, in future studies, more targeted initiatives are needed to help parents develop their knowledge and abilities to give educational support to their children at various stages of schooling. Likewise, programs that promote the parent as teacher model offer the parents a variety of opportunities to learn skills that will help them believe in their own efficacy.

Both parents and teachers will benefit from this research in the future. As a result of this study, parents get understanding and awareness of engaging in activities that result in a more balanced parenting style in order to improve children’s learning outcomes. Moreover, recognizing effective parenting styles can aid in the development of children’s developmental needs, as well as their academic achievement and future professional prospects. Each parenting style has an impact on the social and psychological lives of children. The psychological control is what distinguishes each parenting style from the others. Therefore, it is the responsibility of parents to provide a parental environment and resources that are more conducive to academic success for their children.

The study’s limitations should also be considered because they direct researchers to use these procedures in future research. The current research contributes to a better understanding of the factors that influence children’s learning outcomes. But cross-sectional design of the study is a limitation. Although cross-sectional designs aid in the prediction of relationships, they are unable to capture transitions that may affect the variables’ associations. Therefore, in future researches other methods will be used to further explore these variables.

### Theoretical Implications

This study makes an important contribution to the body of literature. According to the previous studies, parental self-efficacy is defined in the field of parenting as parents’ beliefs about their ability to positively influence their children’s behavior and development. Additionally, parental self-efficacy in schooling can be defined as parents’ beliefs that they can have a positive influence on their children’s learning and academic achievement (Tazouti and Jarlégan, 2019). According to previous research, mothers and fathers with strong parental self-efficacy are more involved in their children’s everyday learning and play activities.

### Practical Implications

The present study includes several implications. Parental involvement plays a significant role in learning outcome of children at educational level. The findings of this study will be helpful for parents in evaluating their parenting styles. It will provide parents an insight to be more capable and eager to become active if they want to effectively affect their children’s education. Besides that, parents’ experiences such as feeling tired, receiving harsh comments and frequently giving in to children’s demands, are all associated to lower parental self-efficacy. These are the factors that should be consider while providing training and awareness session to the parents. Furthermore, when parents are involved as a resource for academic activities at home, the connection between the school and home environments is strengthened. As a result, the child feels more capable of mastering academic tasks at school. Therefore, parents can help their children learn new content by assisting them in scaffolding new concepts. When children see their parents as role models and trusted learning partners, they are better able to appraise their own talents and performance.

This study will not only prove beneficial that parental support provides a sense of security and comfort in an unpredictable culture as the child strives for growth and self-development. Also, effective when parents are involved, they may establish limits, encourage their children, and provide resources as they face the academic, social, and personal obstacles that each day brings. Moreover, when parents go to parent-teacher conferences, open houses, and other school events, they show that they care about their children.

Understanding social learning theory and how to apply it to self-efficacy development through regulating exposure to sources of influence can be extremely beneficial to practitioners. Furthermore, practitioners can increase local parenting support by adopting practices that are congruent with the establishment of environments rich in positive sources of self-efficacy by developing an awareness of parental self-efficacy experiences in a community. This kind of behavior could affect how parents and children interact with each other and, in turn, how children and communities grow and change over time. Lastly, this research can also support future researches as it provides a new perspective to the relationship between parenting styles, learning outcome and parental self-efficacy among Chinese students.

### Limitations

The following are the study’s limitations. First, data from both parents in the family was unavailable, making it unable to run more complicated models involving both parents and make within-family comparisons. More research in this area and the addition of child outcomes would help us learn more about how family relationships affect how children grow and develop. In addition, the sample size was small, limiting the generalizability of the present study.

### Future Suggestions

The recommendations for future researchers are listed below. First, the schools can help parents create a welcoming and comfortable learning environment for their children. Besides that, teachers and schools should strengthen their control and warmth with students in order to drive children to improve their academic performance. Second, it is envisaged that future studies will be able to explore the aspects other than the person and their family context that influence learning outcomes, such as peer group and school environment. Third, future research will include qualitative researches with students to go deeper into the subject and also examine the relationship of study variables with demographics.

Moreover, research is needed to determine how mothers’ and fathers’ working hours and employment conditions affect their participation in a variety of play, learning, and caregiving activities with their children. As a result, future parenting programs could focus on raising parents’ understanding of the need for both parents’ involvement in expressive activities and mentoring/advising duties. This could help them boost their parenting self-efficacy even more. Lastly, academic progress is associated to parenting involvement in a significant way. In this context, additional study on parenting styles, learning outcomes, and parental self-efficacy across cultures is needed to examine the differences in parenting styles. Furthermore, the use of a longitudinal study would be beneficial in analyzing changes in people’s perceptions of their parents and different parenting styles over time.

## Data Availability Statement

The raw data supporting the conclusions of this article will be made available by the authors, without undue reservation.

## Ethics Statement

Ethical review and approval was not required for the study on human participants in accordance with the local legislation and institutional requirements. Written informed consent from the patients/participants or patients/participants legal guardian/next of kin was not required to participate in this study in accordance with the national legislation and the institutional requirements.

## Author Contributions

CK wrote the manuscript. FY performed review editing and submission. Both authors contributed to the article and approved the submitted version.

## Conflict of Interest

The authors declare that the research was conducted in the absence of any commercial or financial relationships that could be construed as a potential conflict of interest.

## Publisher’s Note

All claims expressed in this article are solely those of the authors and do not necessarily represent those of their affiliated organizations, or those of the publisher, the editors and the reviewers. Any product that may be evaluated in this article, or claim that may be made by its manufacturer, is not guaranteed or endorsed by the publisher.
